# Orf Virus Detection in the Saliva and Milk of Dairy Goats

**DOI:** 10.3389/fmicb.2022.837808

**Published:** 2022-03-30

**Authors:** Wentao Ma, Ming Pang, Xinyu Lei, Zishuo Wang, Hao Feng, Shaofei Li, Dekun Chen

**Affiliations:** Veterinary Immunology Laboratory, College of Veterinary Medicine, Northwest Agriculture and Forestry University, Yangling, China

**Keywords:** Orf, Orf virus, saliva, milk, transmission

## Abstract

Orf is a zoonotic and highly contagious disease caused by Orf virus (ORFV) infection. Orf outbreaks in sheep and goats usually lead to high culling rate and mortality in newborn kids and lambs, posing a great threat to the development of goat and sheep industry. Human Orf occurs *via* direct contact with infected animals or fomites. While this disease is traditionally thought to spread through direct contact, whether other transmission routes exist remains unclear. Herein, we report the detection of ORFV in the saliva and milk of dairy goats without clinical Orf symptoms. Further analyses showed that these ORFV are infectious, as they can induce characteristic cytopathic changes in primary mammary and lip cells. Importantly, these ORFV can induce typical Orf lesions after inoculation in ORFV-free dairy goats. This is the first study showing that live, infectious ORFV can be isolated from the saliva and milk of asymptomatic goats, highlighting novel potential transmission routes of ORFV. These findings provide a novel idea for the prevention and control of Orf spread.

## Introduction

Orf virus (ORFV) is the prototype member of the genus *Parapoxvirus* of the family *Poxviridae* (Gelaye et al., 2016; Yao et al., 2020). Infection of this virus causes Orf (also known as contagious pustular dermatitis) occurring primarily in goats and sheep (Spyrou and Valiakos, 2015). A typical course of Orf includes initial erythematous spots in the affected sites, further developing into papules, vesicles and pustules with uncreative exudate as the disease progresses, and finally becoming dry scabs without scar formation (Spyrou and Valiakos, 2015; Bala et al., 2019). While these pathological lesions can be found most frequently in the oral cavity (lip mucosa, tongue, gums), skin of the face and nose, they are also observed at the udder skin of ewes and the testis of male goats (Spyrou and Valiakos, 2015). Orf is a highly contagious and fairly common disease of goats and sheep with worldwide distribution. Although this disease is usually self-limiting that could resolve within 1 month in adult animals, it can be fatal in kids because of their high culling rate and mortality, causing considerable economic losses worldwide every year (Spyrou and Valiakos, 2015).

Orf is also a zoonotic disease which can be transmitted to humans, posing a great risk to public health (Nougairede et al., 2013; Andreani et al., 2019; Kassa, 2021; Qiu and Yanes, 2021). Human cases of Orf have been reported many times and distributed worldwide. People become infected most commonly after contact with diseased animals or contaminated animal products, and the transmission process could be much easier when trauma was present on the skin (Nougairede et al., 2013; Kitchen et al., 2014; Zhang et al., 2014; Hasheminasab et al., 2016; Lopez-Cedeno et al., 2018; Andreani et al., 2019; Qiu and Yanes, 2021). Human-to-human transmission also exists, although such cases are very rare (Turk et al., 2014; Rajkomar et al., 2016). The clinical manifestation of human Orf usually starts as small papules, which then progresses into severe pustular dermatitis lesions (Spyrou and Valiakos, 2015; Qiu and Yanes, 2021; Khan et al., 2022). The lesions are most commonly seen on hands and arms in humans, while they have also been reported on the nose (Gurel et al., 2002; Ata et al., 2017), in perineal locations (Kennedy and Lyell, 1984) and on the forehead (Turk et al., 2014). For most people, Orf is a self-limiting disease and can heal in 3–6 weeks. However, very large lesions and long term morbidity which do not regress spontaneously can be seen in immunocompromised patients (Degraeve et al., 1999; Ara et al., 2008).

Elucidating the transmission route of infectious diseases is important for the development of effective preventative measures. Considering the transmission of Orf, a long-held and widely accepted opinion is that the spread of this disease is done through direct contact with infected animals. According to this opinion, when a healthy animal is feeding on dry hay or other hard fodders, abrasions or cuts can develop around the oral cavity, thus allowing ORFV to enter and establish in the epidermal cells of a healthy animal if it happens to come into contact with a diseased one (Spyrou and Valiakos, 2015; Bala et al., 2019). However, this transmission route of ORFV may not be the only one due to several observations. First, during the outbreak of Orf, many goats or even the total herd can develop Orf without any skin injuries. Second, direct contact may be too inefficient for Orf to spread in a goat herd in a very short period of time, a phenomenon usually observed among dairy goats. Third, newborn kids often develop Orf within the first 2 weeks after birth, a stage when then can only suckle milk, ruling out the possibility to get hurt by hay or other fodders. Fourth, the baby of a ewe without any clinical manifestations of Orf can also develop severe Orf without any contact with other goats. Thus, other transmission pathways of Orf possibly exist, and finding out novel ways in which ORFV spreads is urgently needed to elucidate the mechanism of ORFV infection and for the effective control of this fast-spreading disease.

Taking into account that the pathological lesions of Orf goats are generally present in areas of the mouth cavity and the udder, and it is not uncommon for viruses to transmit through saliva and milk (Abrahao et al., 2009; Clarke et al., 2018; Ferri and Vergara, 2021), we focused on saliva and milk of dairy goats and evaluated whether infectious ORFV are present in these two sites. The results showed that live ORFV can be detected in the saliva and milk of dairy goats. Importantly, these ORFV are pathogenic both *in vitro* and *in vivo*. This is the first study reporting the detection and isolation of ORFV in saliva and milk, highlighting novel potential transmission routes of this virus in dairy goats.

## Materials and Methods

### Sample Collection

Female Guanzhong dairy goats of 2–3-year-old without Orf clinical symptoms were randomly selected from local farms. Three herds were examined. Herds were selected because they experienced orf outbreaks in recent years. Farm 1 and Farm 2 was experiencing clinical orf in animals when sampled. Farm 3 last experienced clinical orf in the spring of 2020. Blood was collected from the jugular vein of goats (Cheng et al., 2018). For saliva collection, sterile oral swabs were placed upon and gently stirred around the tongue of the goats for a few seconds. The swabs were then put into sterile saline for further analysis. For milk collection, the first 3 streams of milk were discarded prior to collection. In total 136 blood samples, 119 saliva samples and 103 milk samples were collected in this study. All samples were collected in accordance with the protocols of the Research Ethics Committee of Northwest A&F University.

### PCR Amplification, Phylogenetic, and Nucleotide Homology Analyses

Viral DNA was extracted using a DNA extraction kit according to the manufacturer’s manual (Tiangen Biotech, Beijing, China). The extracted DNA was subjected to PCR amplification for the detection of ORFV. Primers were designed based on the conserved *B2L* sequence of ORFV: forward (5′-CGGAATTCAGTCCGCGAAGAAGTTTTTG-3′) and reverse (5′-CCCTCGAGGCGAGTCCGAGAAGAATACG -3′), and the variable *ORF128* sequence of ORFV: forward (5′- CGG AATTCTTGCCACCATGGACTTTCTAGG-3′) and reverse (5′- CGGGATCCAAATACTCGGCCAGTC -3′). A total reaction volume of 25 μL containing 12.5 μL of 2 × Taq MasterMix (CWbiotech, Jiangsu, China), 0.5 μL of each primer, 2 μL of DNA template and 9.5 μL of ddH_2_O was used for PCR amplification. The thermal cycling process was the same as we reported before (Tong et al., 2020; Tang et al., 2021). PCR products were analyzed by 1.5% agarose gel electrophoresis. The PCR products were sequenced in Sangon Biotech (Shanghai, China). The gene sequences were compared with other available ORFV *B2L* or *ORF128* complete gene sequences by BLAST programs in the GenBank database. A phylogenetic tree was constructed by a maximum likelihood method with MEGA software (Pennsylvania State University, PA, United States). The deduced amino acid sequences were analyzed using a DNAStar software (Madison, WI, United States).

### Preparation of Goat Primary Mammary, Lip and Testicular Cells

For primary cell isolation, the relative tissue of a lactating Guanzhong dairy goat was obtained from a local slaughter-house immediately after slaughter. The tissue was wiped with 75% ethanol and processed under aseptic conditions. The tissue was further cut into 1 mm^3^ pieces and digested with 1 mg/mL of collagenase IV (Gibco, Thermo Fisher Scientific, Waltham, MA, United States) at 37°C with 5% CO_2_ for 12 h. The digested tissues were filtered by 200-mesh nylon filter cloth screens and washed twice with serum-free DMEM/F12 medium (Hyclone, Thermo Fisher Scientific, Waltham, MA, United States). The cells were resuspended with DMEM/F12 medium with 10% fetal bovine serum (Gibco) and 1% Penicillin-Streptomycin (10,000 units) (Hyclone) and cultured for further use.

### Orf Virus Isolation and *in vitro* Infection of Cells

For milk ORFV isolation, ORFV-positive milk was centrifuged at 850 *g* for 10 min and the whey at the lower layer that contained ORFV was collected. For saliva ORFV isolation, ORFV-positive oral swabs were soaked in sterile 0.9% NaCl solution at 4°C for 24 h. The collected ORFV-containing whey and NaCl solution were further filtered through 0.45 and 0.22 μm filters. The isolated goat primary cells were collected and incubated with the aforementioned ORFV-containing filtrates for 1 h at 37°C with 5% CO_2_. Then, the cells were supplemented with 10 mL of Minimum Essential Medium (MEM, Gibco) with 2% fetal bovine serum (Gibco). Typical cytopathic effect (CPE) was observed after five generations of culture.

### Experimental Infection of Goats With Milk and Saliva Orf Virus

For animal infection experiment, the inner thighs of a goat were scratched 3–5 places with a sterile syringe needle. 1 mL of ORFV (in MEM, 10^5^.^6^/1 mL TCID50) isolated from the milk or saliva was applied to each scratch using cotton swabs. Control goats were inoculated with 1 mL of MEM at the scratches. The scratch lesions were monitored daily for 10 days. The study was performed in accordance with the protocols of the Research Ethics Committee of Northwest A&F University.

## Results

### Orf Virus Is Present in the Saliva and Milk of Goats

**FIGURE 1:**
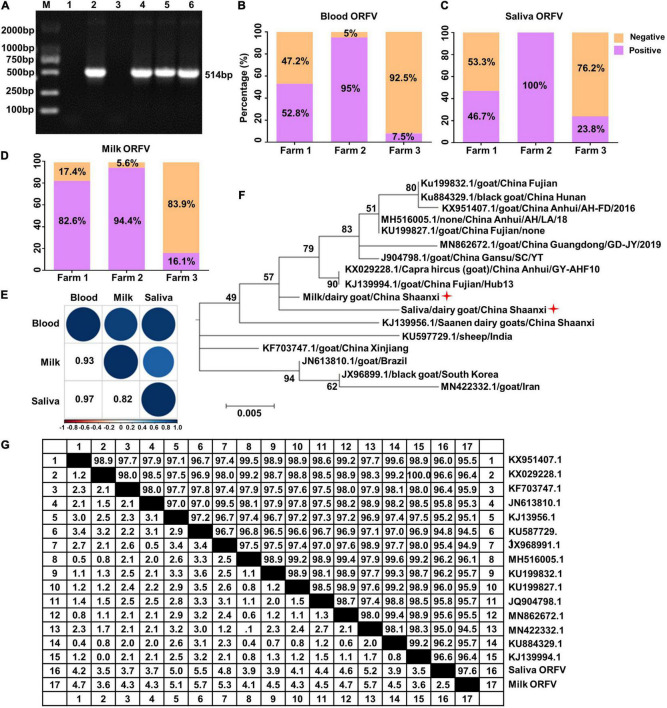
Detection of ORFV in the saliva and milk of dairy goats. **(A)** Representative PCR results of ORFV *B2L* gene. M, DNA marker. Lane 1, negative control using double-distilled water instead of DNA template. Lane 2, positive control with DNA extracted from purified ORFV. Lane 3, DNA extracted from blood of ORFV negative dairy goat. Lane 4, ORFV from the peripheral blood of dairy goats. Lane 5, ORFV from the saliva of dairy goats. Land 6, ORFV from the milk of dairy goats. **(B–D)** Detection rates of ORFV from the peripheral blood, saliva and milk of dairy goats from three local farms. **(E)** Pearson’s correlation coefficient analysis based on ORFV detection rate of the peripheral blood, saliva and milk of dairy goats from the three farms. **(F)** Phylogenetic tree comparing *B2L* sequences of the saliva and milk ORFV isolated here (indicated by a red star) and other published *B2L* sequences. **(G)** Similarity comparison of the deduced B2L sequences of saliva and milk ORFV at the amino acid level with other published B2L sequences.

We previously reported that infectious ORFV could be isolated from the peripheral blood of dairy goats (Cheng et al., 2018). To investigate whether ORFV can be detected in the saliva and milk of dairy goats and the relationship of these ORFV with blood ORFV, we randomly collected 136 peripheral blood samples, 119 saliva samples and 103 milk samples of dairy goats of three different farms. PCR result confirmed the presence of ORFV in the peripheral blood, saliva and milk of dairy goats (Figure 1A), with overall ORFV detection rates being 32.4, 53, and 44.7% in these three types of samples (Tables 1–3). Specifically, ORFV detection rate in the peripheral blood of dairy goats of these three farms was 52.8, 95, and 7.5%, respectively (Figure 1B). For saliva samples, ORFV detection rates were 46.7, 100, and 23.8%, respectively (Figure 1C). The rates for milk samples were 82.6, 94.4, and 16.1%, respectively (Figure 1D). Interestingly, correlation analysis indicated that ORFV positivity in the peripheral blood, saliva and milk were strongly positively correlated since the Pearson’s correlation coefficient for the blood and saliva was 0.97, for the blood and milk was 0.93, and for the milk and saliva was 0.82 (Figure 1E). The phylogenetic analysis based on the *B2L* and *ORF128* genes showed that the ORFV isolated here had the closest evolutionary relationship with one isolated in Fujian Province, China (Figure 1F) and one isolated in Germany (Supplementary Figure 1A), respectively. In addition, the *B2L* and *ORF128* sequences of saliva and milk ORFV showed 94.5–100.0%, and 94.9–99.9% similarity at the amino acid level with other published ORFV strains (Figure 1G and Supplementary Figure 1B).

**TABLE 1:** Detection rate of ORFV from the peripheral blood of dairy goats.

Farms	No. of samples	No. and% of positive
Farm 1	36	19 (52.8%)
Farm 2	20	19 (95.0%)
Farm 3	80	6 (7.5%)
Total	136	44 (32.4%)

**TABLE 2:** Detection rate of ORFV from the saliva of dairy goats.

Farms	No. of samples	No. and% of positive
Farm 1	30	14 (46.7%)
Farm 2	20	20 (100%)
Farm 3	80	19 (23.8%)
Total	130	44 (53.0%)

**TABLE 3:** Detection rate of ORFV from the milk of dairy goats.

Farms	No. of samples	No. and% of positive
Farm 1	23	19 (82.6%)
Farm 2	18	17 (94.4%)
Farm 3	62	10 (16.1%)
Total	103	46 (44.7%)

### *In vitro* Evidence Confirming the Pathogenicity of the Orf Virus Isolated From the Saliva and Milk

**FIGURE 2:**
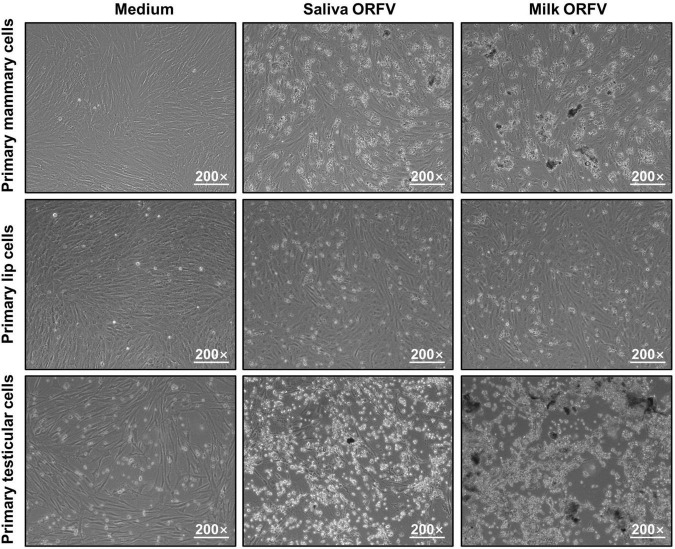
Infection of permissive cells by ORFV isolated from the saliva and milk of dairy goats. Goat Primary lip cells, mammary cells and testicular cells were cultured and infected with ORFV (10^5^.^6^/1 mL TCID50) isolated from the saliva or milk of dairy goats. Control cells were treated with MEM of the same volume. 72 h later, the cells were observed under a light microscope.

To determine whether the saliva- and milk-isolated ORFV are live virus that could actively infect permissive cells, these viruses were applied to *in vitro*-cultured ORFV-free goat primary lip, mammary and testicular cells (Supplementary Figure 2A). As a result, CPE including rounding, loss of adherence and lysis of the infected cells were induced, suggesting that these ORFV present in the saliva and milk of dairy goats are infectious (Figure 2).

### *In vivo* Evidence Confirming the Pathogenicity of the Orf Virus Isolated From the Saliva and Milk

**FIGURE 3:**
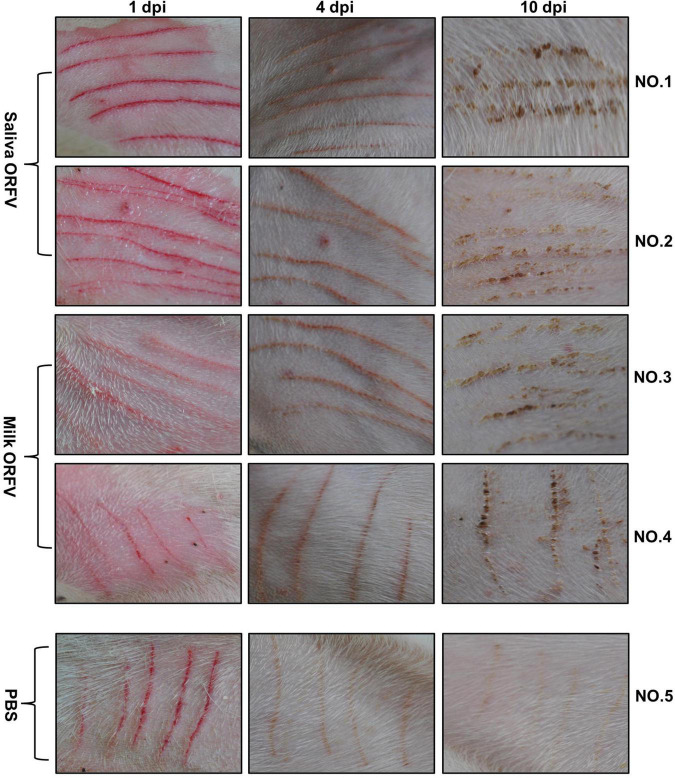
Experimental infection of dairy goats using saliva- and milk-isolated ORFV. ORFV-free dairy goats (NO. 1–5) were scratched with sterile syringe needles in the inner thighs and inoculated with saliva ORFV (Saliva ORFV), milk ORFV (Milk ORFV), or MEM (Medium). The scratch lesions were monitored daily for 10 days. The results are shown for 1 day (1 dpi), 4 days (4 dpi), and 10 days (10 dpi) post infection.

To confirm that ORFV isolated from saliva and milk are pathogenic *in vivo*, healthy dairy goats were inoculated in the inner thighs with medium or saliva or milk ORFV. While the wounds of medium-treated goats recovered rapidly without any manifestations of Orf symptoms, both saliva ORFV- and milk ORFV-challenged wounds showed typical Orf symptoms such as papules, vesicles, and crusty scabs (Figure 3 and Supplementary Figure 2B). These results showed that saliva and milk ORFV was capable of causing clinical symptoms of goat Orf *in vivo*.

## Discussion

Orf is a very common disease of dairy goats, particularly affecting the health of newborn kids who are very sensitive to ORFV and the culling rate and mortality could reach to 90% (Zhao et al., 2010; Spyrou and Valiakos, 2015). According to others’ reports and our investigations, many kids develop clinical Orf symptoms within the first 2 weeks after birth (Spyrou and Valiakos, 2015). The outbreak of Orf could be very quick and spread extremely rapidly in kids, whose antiviral immunity are overall relatively week and suffer greatly from the pathological lesions especially during suckling milk (Spyrou and Valiakos, 2015). In addition, when Orf affects the udder or the teats of a lactating ewe, the painful lesions as well as secondary infections of the udder (mastitis) usually rendered the ewe less willing to milk her kids and even abandon them if the lesions continue or worsen. Thus, the widespread outbreak of Orf has caused great economic loss on goat industry, especially because of its fatal impact on newborn kids. According to the present study, infectious ORFV can be detected in the milk of lactating goats. This finding strongly suggests that milk can be a possible route for Orf transmission, and suckling milk by newborn kids is likely one of the most important reasons for the rapid outbreak of Orf in kids.

In many dairy goat farms of China, kids are kept together with their mothers after birth and suckle milk directly from the udder of their mothers. Coincidentally, prevalence of Orf in newborn kids can be very severe in such farms (data not shown). Combined with the finding of the present study, milk-mediated transmission of ORFV from mothers to newborn kids may be a major cause of this phenomenon, and viral particle positivity in the milk should be considered as an important risk factor of kids Orf. In fact, mother-to-child transmission *via* milk is not uncommon for a wide range of viruses such as human immunodeficiency virus (Suarez et al., 2019), Zika virus (Sotelo et al., 2017), Ebola virus (Medina-Rivera et al., 2021), bovine vaccinia virus (Abrahao et al., 2009), bovine leukemia virus (Barzegar et al., 2021), tick-borne encephalitis virus (Ronai and Egyed, 2020) and peste des petits ruminants virus (Clarke et al., 2018). Bearing this in mind, another feeding style of goats under which the kids are kept separately from their mothers and regularly given pasteurized milk collected together from all the lactating goats, which style is also adopted by many goat farms of China, should thus be encouraged. In fact, pasteurization and heat treatment of the milk could effectively inactivate a wide range of milk-borne viruses and thus abrogate the further transmission of the viruses (Pearce et al., 2012; Donalisio et al., 2018; Groner et al., 2018).

The presence of infectious ORFV in the milk of dairy goats draws attention to a potential risk to public health, as raw milk consumption is still common in rural populations of China. Although it remains unclear whether ORFV can reach humans *via* goat milk, alimentary transmission of viruses after consuming unthoroughly heated milk has so far been reported in several cases (Maschmann et al., 2001; Brockmann et al., 2018). Therefore, avoiding the direct consumption of fresh or unpasteurized milk should be highly encouraged to prevent these food-borne illnesses. In addition, farm workers and consumers should be very careful when milking the goats and when touching fresh milk to minimize the potential risk of such infections. To this end, protective measures (i.e., wearing protective clothing and gloves) should be taken when touching fresh milk.

Another finding of the present study is that live ORFV can be isolated from the saliva of goats. For dairy goats, an important maternal behavior is that the ewe will lick the newborn kids immediately after lambing. Usually, the licking cleaning begins with the mouth and nose first, followed by the face, the head and other parts of the newborn kids. While this behavior is necessary for the establishment of the mother-infant bond (Nagasawa et al., 2012), it could inevitably mediate the transmission of saliva-borne diseases. Considering the finding of this study, licking the newborn kids should be an unignored explanation of kid Orf if their mother happens to carry ORFV in the saliva. In addition, lick blocks are commonly used in many dairy goat farms for the supplementation of minerals and trace elements (Banchero et al., 2009). Although direct evidence is lacking, these lick blocks can possibly serve as reservoirs of ORFV and allow the spread of Orf to goat housed together. The same conclusion may be also applied to the transmission of Orf through the fence, the wall, the waterers, the feeders and other shared devices of a farm which could be licked by ORFV-carrying goats.

Because infectious ORFV can be present in the saliva of dairy goats, saliva droplet transmission may also be a possible route for Orf spread when goats were kept in close contact due to excessive herd density. In addition, goats have the nature to crowd together. All these aspects could promote the potential transmission of Orf *via* saliva droplets. However, further evidence is needed in the future to support this conclusion.

For the transmission of ORFV to humans, the most widely reported route is direct contact with diseased animals (Nougairede et al., 2013; Kitchen et al., 2014; Zhang et al., 2014; Hasheminasab et al., 2016; Lopez-Cedeno et al., 2018; Andreani et al., 2019; Qiu and Yanes, 2021; Khan et al., 2022). However, several important clues doses implicate the presence of other transmission routes. For example, Khan et al. (2022) reported a case of Orf in a Scottish Sheep farmer with pain and discomfort. She had not seen any Orf lesions around the mouths of her sheep. In such case, ORFV may be likely transmitted to the patients through the contact with devices of the farm contaminated by ORFV-carrying asymptomatic sheep. In consistent with this hypothesis, another report showed that ORFV could be transmitted from human to human *via* the common use of tweezers (Turk et al., 2014). In addition, supporting the findings of the present study that milk ORFV can be infectious, Hasheminasab et al. (2016) reported that a human case of Orf was associated with milking of sheep under poor hygiene conditions.

In summary, the present study shows that infectious ORFV can be isolated from the saliva and milk of dairy goats. These findings suggest that several saliva-borne and milk-borne transmission routes of Orf possibly exist and provide a novel idea for the prevention and control of Orf spread.

## Data Availability Statement

The original contributions presented in the study are included in the article/Supplementary Material, further inquiries can be directed to the corresponding author.

## Ethics Statement

The animal study was reviewed and approved by the Research Ethics Committee of Northwest A&F University.

## Author Contributions

WM and DC designed the research. WM, MP, XL, ZW and SL performed the experiments. MP and HF analyzed the data. WM and MP wrote and revised the manuscript. All authors contributed to the article and approved the submitted version.

## Conflict of Interest

The authors declare that the research was conducted in the absence of any commercial or financial relationships that could be construed as a potential conflict of interest.

## Publisher’s Note

All claims expressed in this article are solely those of the authors and do not necessarily represent those of their affiliated organizations, or those of the publisher, the editors and the reviewers. Any product that may be evaluated in this article, or claim that may be made by its manufacturer, is not guaranteed or endorsed by the publisher.
