# Supplementary material for: Orf Virus Detection in the Saliva and Milk of Dairy Goats
Source: Front Microbiol. 2022 Mar 30;13:837808. doi: 10.3389/fmicb.2022.837808 (PMC9006325; doi:10.3389/fmicb.2022.837808)
Supplement: Supplementary file 1 [file Presentation_1.PPTX]

## Slide 1
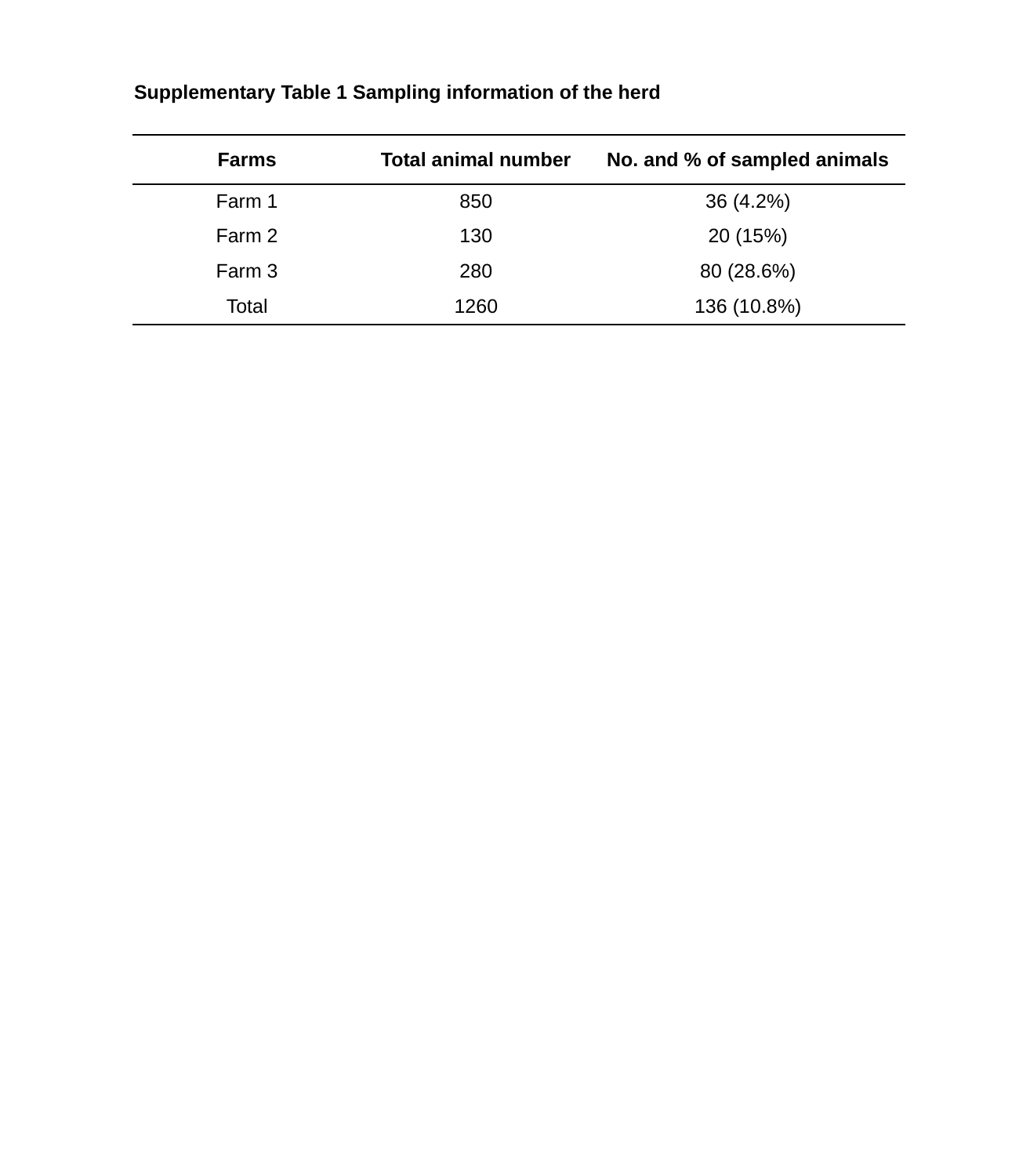

Supplementary Table 1 Sampling information of the herd
| Farms | Total animal number | No. and % of sampled animals |
| --- | --- | --- |
| Farm 1 | 850 | 36 (4.2%) |
| Farm 2 | 130 | 20 (15%) |
| Farm 3 | 280 | 80 (28.6%) |
| Total | 1260 | 136 (10.8%) |

## Slide 2
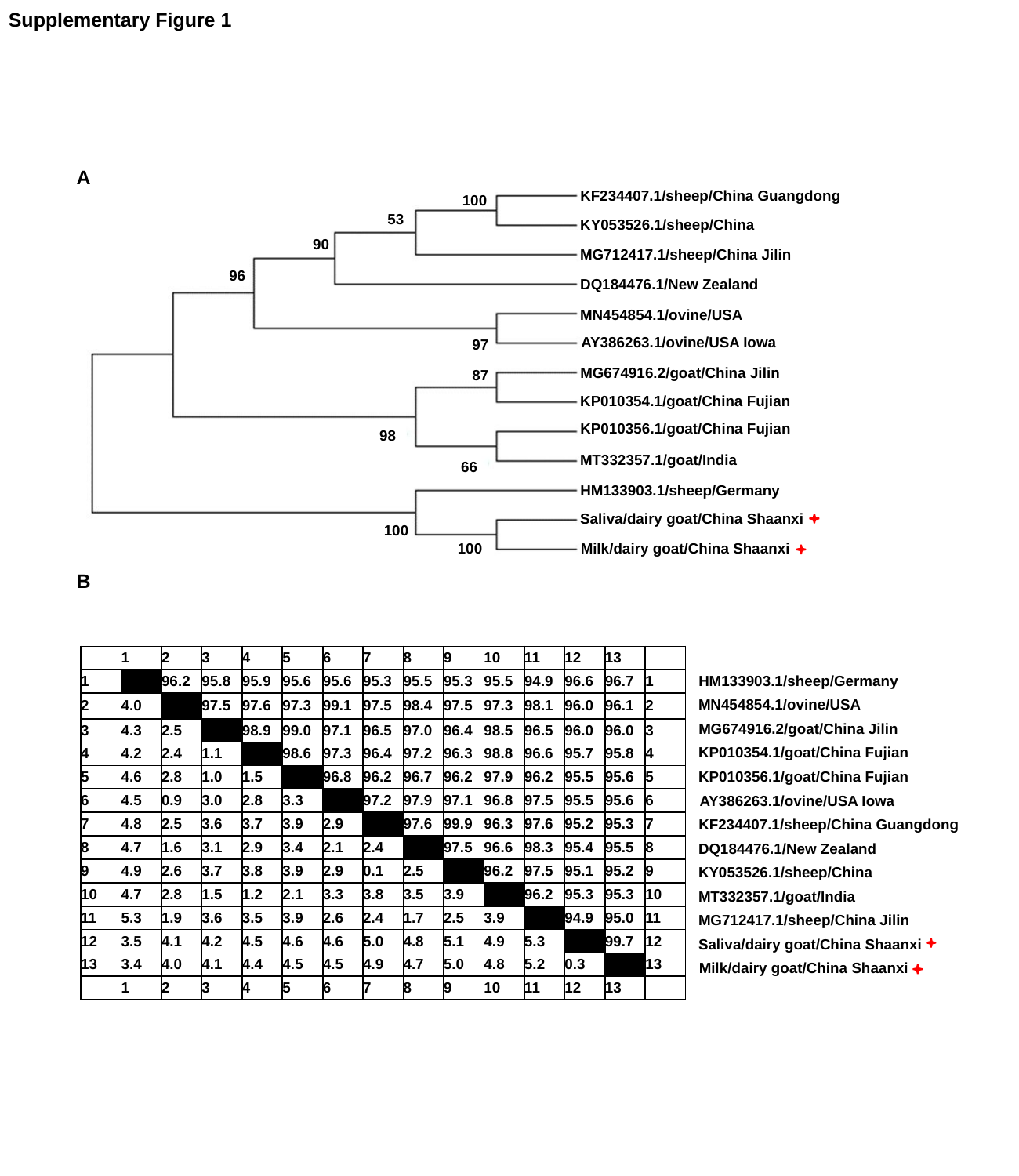

Supplementary Figure 1
A
KF234407.1/sheep/China Guangdong
100
53
KY053526.1/sheep/China
90
MG712417.1/sheep/China Jilin
96
DQ184476.1/New Zealand
MN454854.1/ovine/USA
AY386263.1/ovine/USA Iowa
97
MG674916.2/goat/China Jilin
87
KP010354.1/goat/China Fujian
KP010356.1/goat/China Fujian
98
MT332357.1/goat/India
66
HM133903.1/sheep/Germany
Saliva/dairy goat/China Shaanxi
100
100
Milk/dairy goat/China Shaanxi
B
| | 1 | 2 | 3 | 4 | 5 | 6 | 7 | 8 | 9 | 10 | 11 | 12 | 13 | |
| --- | --- | --- | --- | --- | --- | --- | --- | --- | --- | --- | --- | --- | --- | --- |
| 1 | | 96.2 | 95.8 | 95.9 | 95.6 | 95.6 | 95.3 | 95.5 | 95.3 | 95.5 | 94.9 | 96.6 | 96.7 | 1 |
| 2 | 4.0 | | 97.5 | 97.6 | 97.3 | 99.1 | 97.5 | 98.4 | 97.5 | 97.3 | 98.1 | 96.0 | 96.1 | 2 |
| 3 | 4.3 | 2.5 | | 98.9 | 99.0 | 97.1 | 96.5 | 97.0 | 96.4 | 98.5 | 96.5 | 96.0 | 96.0 | 3 |
| 4 | 4.2 | 2.4 | 1.1 | | 98.6 | 97.3 | 96.4 | 97.2 | 96.3 | 98.8 | 96.6 | 95.7 | 95.8 | 4 |
| 5 | 4.6 | 2.8 | 1.0 | 1.5 | | 96.8 | 96.2 | 96.7 | 96.2 | 97.9 | 96.2 | 95.5 | 95.6 | 5 |
| 6 | 4.5 | 0.9 | 3.0 | 2.8 | 3.3 | | 97.2 | 97.9 | 97.1 | 96.8 | 97.5 | 95.5 | 95.6 | 6 |
| 7 | 4.8 | 2.5 | 3.6 | 3.7 | 3.9 | 2.9 | | 97.6 | 99.9 | 96.3 | 97.6 | 95.2 | 95.3 | 7 |
| 8 | 4.7 | 1.6 | 3.1 | 2.9 | 3.4 | 2.1 | 2.4 | | 97.5 | 96.6 | 98.3 | 95.4 | 95.5 | 8 |
| 9 | 4.9 | 2.6 | 3.7 | 3.8 | 3.9 | 2.9 | 0.1 | 2.5 | | 96.2 | 97.5 | 95.1 | 95.2 | 9 |
| 10 | 4.7 | 2.8 | 1.5 | 1.2 | 2.1 | 3.3 | 3.8 | 3.5 | 3.9 | | 96.2 | 95.3 | 95.3 | 10 |
| 11 | 5.3 | 1.9 | 3.6 | 3.5 | 3.9 | 2.6 | 2.4 | 1.7 | 2.5 | 3.9 | | 94.9 | 95.0 | 11 |
| 12 | 3.5 | 4.1 | 4.2 | 4.5 | 4.6 | 4.6 | 5.0 | 4.8 | 5.1 | 4.9 | 5.3 | | 99.7 | 12 |
| 13 | 3.4 | 4.0 | 4.1 | 4.4 | 4.5 | 4.5 | 4.9 | 4.7 | 5.0 | 4.8 | 5.2 | 0.3 | | 13 |
| | 1 | 2 | 3 | 4 | 5 | 6 | 7 | 8 | 9 | 10 | 11 | 12 | 13 | |
HM133903.1/sheep/Germany
MN454854.1/ovine/USA
MG674916.2/goat/China Jilin
KP010354.1/goat/China Fujian
KP010356.1/goat/China Fujian
AY386263.1/ovine/USA Iowa
KF234407.1/sheep/China Guangdong
DQ184476.1/New Zealand
KY053526.1/sheep/China
MT332357.1/goat/India
MG712417.1/sheep/China Jilin
Saliva/dairy goat/China Shaanxi
Milk/dairy goat/China Shaanxi

## Slide 3
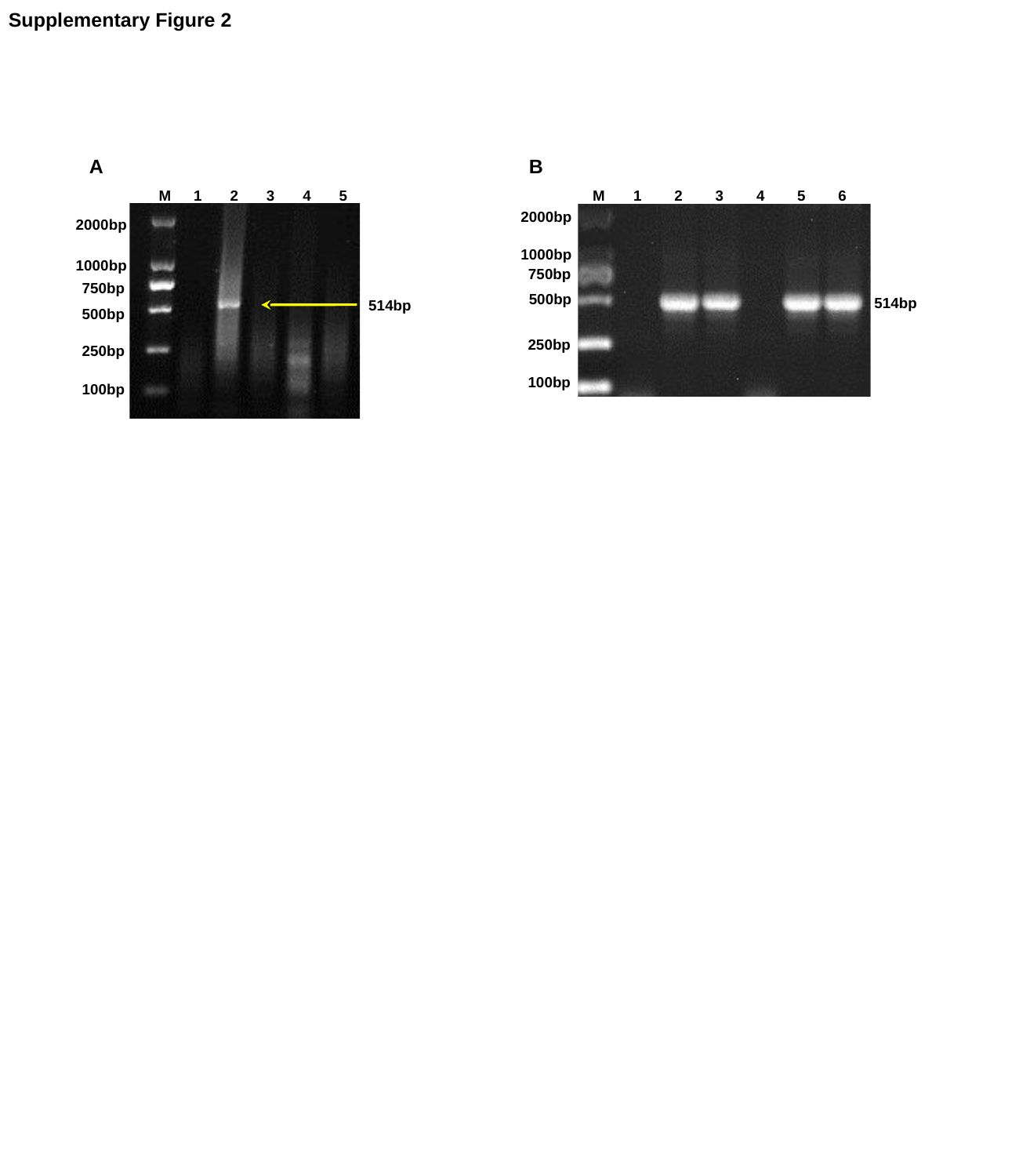

Supplementary Figure 2
A
B
M
4
5
6
1
2
3
4
5
M
1
2
3
2000bp
2000bp
1000bp
1000bp
750bp
750bp
500bp
514bp
514bp
500bp
250bp
250bp
100bp
100bp
