# Supplementary material for: Orf Virus Detection in the Saliva and Milk of Dairy Goats
Source: Front Microbiol. 2022 Mar 30;13:837808. doi: 10.3389/fmicb.2022.837808 (PMC9006325; doi:10.3389/fmicb.2022.837808)
Supplement: Supplementary file 2 [file Data_Sheet_1.docx]

**Supplementary Figure 1 Sequences analysis of variable orf128 gene of ORFV.**

(A) Phylogenetic tree comparing orf128 sequences of the saliva and milk ORFV isolated here (indicated by a red star) and other published orf128 sequences. (B) Similarity comparison of the deduced orf128 sequences of saliva and milk ORFV at the amino acid level with other published orf128 sequences of ORFV complete gene.

**Supplementary Figure 2 Representative PCR results of ORFV *B2L* gene.**

(A) Goat primary lip, mammary and testicular cells were screened for ORFV before they were used for ORFV infection. M, DNA marker. Lane 1, negative control using double-distilled water instead of DNA template. Lane 2, positive control. Lane 3, goat primary lip cells. Lane 4, goat primary mammary cells. Lane 5, goat primary testicular cells. (B) The scars or peripheral blood of dairy goats treated as described in Figure 3 were used and analyzed. M, DNA marker. Lane 1, negative control using double-distilled water instead of DNA template. Lane 2, DNA extracted from the scars of goat No.1 in Figure 3. Lane 3, DNA extracted from the scars of goat No.2 in Figure 3. Lane 4, DNA extracted from the blood of goat No.5 in Figure 3. Lane 5, DNA extracted from the scars of goat No.3 in Figure 3. Lane 6, DNA extracted from the scars of goat No.4 in Figure 3.
